# Development of one part sustainable alkali activated binder system using slag, flyash and micro calcined kaolin

**DOI:** 10.1038/s41598-026-46876-1

**Published:** 2026-04-07

**Authors:** M. G. Girish, Shreelaxmi Prashant, H. M. Jagadisha, Sinchana Prabha

**Affiliations:** Manipal Institute of Technology, Manipal Academy of Higher Education, Manipal, India

**Keywords:** Box-Behnken design, One-part geopolymer, Response Surface method, Alkali-activated binder, Ternary blend mix, Optimisation, Engineering, Materials science

## Abstract

A one-part alkali-activated binder system is a sustainable, low-environmental-footprint binder. However, optimising this binder using industrial by-products for reliable performance remains a challenge. Conventional cement production is a major source of global CO₂ emissions, challenging the achievement of the Paris Agreement targets and net-zero goals by 2050. To align with Sustainable Development Goals 9 and 11, the development and adoption of low-carbon alternative binder materials is essential for sustainable infrastructure. This study investigates the formulation and optimisation of a novel One-Part Ternary Alkali-activated Binder (OP-TAB) using Box–Behnken Response Surface Methodology (RSM). A ternary blend consists of Ground Granulated Blast Furnace Slag (GGBS), flyash (FA) and Micro Calcined Kaolin (MCK) in varying proportions, mixed with solid activators. The properties of the binder depend on the proportions of the precursors, activator dosage and water-to-binder ratio. A three-level Box–Behnken design was employed to evaluate the influence of FA, MCK, and water-to-binder ratio (W/B) on key performance indicators, namely flowability, setting time, and compressive strength. GGBS served as the primary precursor, with sodium metasilicate as the solid activator. Optimisation via desirability function yielded an ideal mix comprising of 23% FA, 24% MCK, 53% GGBS, and a W/B ratio of 0.265. This formulation achieved a flowability of 130.12%, initial and final setting times of 114.58 and 186.47 min, respectively, and compressive strengths of 28.12 MPa at 7 days and 55.45 MPa at 28 days, respectively. Analysis of Variance (ANOVA) is performed to evaluate most significant parameters and accuracy in DOE. Microstructural analysis using Scanning Electron Microscope (SEM), with Energy Dispersive X-ray Spectroscopy (EDS), and X-Ray Diffraction (XRD) confirmed the formation of reaction products consistent with the observed mechanical performance. The proposed one-part binder system exhibits a reduced environmental footprint and lower reliance on cement, offering a sustainable and durable alternative for construction applications while supporting circular economy principles.

## Introduction

The excessive reliance on Ordinary Portland Cement (OPC) in the construction industry has produced substantial environmental and resource-related problems. Each kilogram of OPC production emits approximately 0.66 to 0.82 kg of CO₂, contributing substantially to global greenhouse gas emissions^1^. In addition to emissions, the process also uses large amounts of natural resources, which has led to calls for the development of greener alternatives that do not affect performance. Alkali-activated materials (AAMs) have been identified as promising low-carbon alternatives, which are produced through the activation of aluminosilicate-rich precursors with alkaline solutions such as sodium hydroxide or sodium silicate, often at low thermal activation conditions^2,3^. In order to make a binder suitable for large-scale infrastructure, the selection of precursors needs to be judiciously done by taking into consideration its bulk availability, since most of the infrastructure projects demand concreting in large quantities^4,5^.

While two-part AAM systems, requiring liquid activators, have demonstrated high performance, their practical application is hindered by safety and handling issues. One-part alkali-activated binders (OP-AABs), often referred to as “just-add-water” geopolymers^6^ address these limitations by incorporating solid activators like sodium metasilicate directly into the dry mix safety^7,8^, simplifying on-site usage and improving simplifying on-site usage and improving safety^9^. Amongst the commonly used precursors, GGBS-based AAMs offer a wide range of application which demands high strength and faster setting like roads, runways etc. This boosts the early removal of forms, hence GGBS-based alkali-activated binder systems can be suited for infrastructure projects. GGBS possesses hydraulic as well as pozzolanic properties^10,11^. GGBS is also characterised by the presence of alumino-silicates supporting alkali activation, resulting into formation of a chemically stable binder, mainly consisting of calcium alumino silicate hydrates (CASH). Moreover, GGBS-based AAMs eliminate the need for high-temperature curing, thereby reducing the energy demand for concreting^5,12,13^.

While GGBS-based AAMs are loaded with a wide range of benefits, the quick setting could sometimes cause volume stability issues. GGBS activation is exothermic and hence associated with the release of heat. Because of this, the binder undergoes rapid setting and is susceptible to shrinkage-related issues^14^. On the other hand, flyash, also an industrial by-product from the thermal power plants, mainly comprising of silica and alumina, is suited for the alkali activation. However, it activates slowly, which makes it necessary to use heat curing to boost the reactivity. FA activation predominantly produces NASH, which is chemically stable and produces a durable binder^15,16^. Recent infrastructure demands a binder possessing high strength whose setting characteristics can be controlled and fine-tuned to suit the project requirements. This necessitates the use of a precursor that has slower setting characteristics and improves the shrinkage resistance by dissipating the heat produced during GGBS activation.

FA and GGBS exhibit complementary characteristics. FA contributes to long-term durability but requires curing temperatures of about 60 to 70 °C and has slower setting and strength development^17^, whereas GGBS promotes rapid setting and early strength due to its high calcium oxide content that allows the growth of calcium-alumino-silicate-hydrate (C-A-S-H) gels^18^. Since GGBS is more reactive, the heat generated during activation of GGBS will be used for activation of flyash, when its blended with GGBS. This reduces shrinkage and other related issues and results into a stable. This system produces a stable and chemically inert binder suitable for wide range of infrastructure applications^4,5^.

MCK, yet another aluminosilicate characterised with much finer particle size and high surface area, compared to GGBS and FA^19,20^. This can be used as one of the potential precursors, although its performance may vary depending on its mineral composition and iron oxide content^21,22^. Incorporation of MCK into the binder system not only improves sustainability but also enhances its mechanical performance because of finer particle size and pore filling effect, which is required for high-strength applications^23^. MCK complements with the other ingredients of the binder system by producing additional C-NASH that contributes to not only densifying the system but also mitigating the volume changes issues^24^.

Sodium metasilicate, as a solid activator, enhances workability and strength while eliminating the need for corrosive liquid activators^25,26^. These materials are not only useful in the development of user-friendly binders but also provide synergistic effects, such as enhanced mechanical properties, chemical resistance, and stability^27,28^. However, the complexity arising from the various variables involved, such as the ratio of the precursors, the type and amount of the activator, and the curing conditions, requires a systematic approach for optimization^29^.

The interaction effects between the alkali activation products are mainly responsible for the development of binder properties such as setting times and strength development. Hence, proportioning of precursors play key role in the development of the binder with required fresh and hardened properties. Given the complexity of interactions among multiple variables - precursor ratios, activator dosage, and curing conditions - a systematic optimisation approach is essential. Response Surface Methodology (RSM) enables the development of empirical models by fitting a quadratic surface to experimental data, allowing for the identification of optimal mix proportions with a minimal number of trials^30,31^. RSM techniques, particularly the Box–Behnken Design (BBD), provide a robust statistical framework for modelling and optimising such systems^32,33^. RSM proved to be an effective tool to optimise AAB systems, demonstrating its effectiveness in improving mechanical performance through statistical modelling^34^. Table 1 summarises the previous studies on one-part alkali-activated systems that adopted the Response Surface Methodology (RSM) technique for mix design optimisation and performance evaluation.

**Table 1:** Summary of previous studies on one-part alkali-activated binder system with RSM technique.

Types of composition	DOE Employed	Key findings	Reference
Solid alkali-activated fly ash-based concrete	Response Surface Method	Optimal mix achieved: 11.7% GBFS, 6.9% SF, 1.67% NaOH with prediction errors below 5%	^35^
Alkali activated slag concrete	Response Surface Method	Based on RSM’s D-Optimal, the optimised independent variables, S = 416.418 kg/m3, A/B = 0.488 and F/C = 0.554 are validated with experiment and found that error < 10%	^36^
Alkali-activated slag mortar	Response Surface Method	The optimum values of limestone powder, glass powder, and alkali content with highest desirability of 0.706 are 15.80, 17.02, and 6.71%, respectively. The predicted combination was validated by confirmatory tests and the error in prediction was found to be within 3.5%.	^37^
One-part alkali-activated grouting material	Response Surface Method	Al/Ca ratio most important for 28-day compressive strength, contrary to traditional Si/Al emphasis. Five key mix parameters identified: Si/Al, Al/Ca, Na_2_O/H_2_O, B_2_O_3_/CaO, B_2_O_3_/Al_2_O_3_. Higher Si/Al and Al/Ca ratios deteriorate microstructure and hinder C-A-S-H gel formation	^38^
Alkali-activated pastes	Response Surface Method	The study used RSM to optimize mix proportions, developing quadratic models with high R2 values (0.8843–0.9913) for predicting compressive strength, tensile strength, and water absorption.	^34^

BBD employs a systematic combination of midpoints and edge points of the design space, excluding extreme corner points, thereby improving experimental efficiency and reducing material consumption. The use of BBD also facilitates multi-response optimisation using the desirability function. Alongside, ANOVA can be performed to assess the statistical significance of individual factors and their interactions.

The present study uses the BBD as a statistical tool to optimise a one-part ternary alkali-activated binder system. The optimisation aims to evaluate the combined effect of FA, MCK as partial replacement with GGBS, and W/B ratio on the performance of flowability, setting time, and compressive strength. Analysis of Variance (ANOVA) is performed to check the significance of each variable and its interaction effects. Microanalysis on the binders to help to understand the reaction mechanisms and phase formation. In addition, the sustainability and cost performance of the OP-TAB system are assessed and compared with conventional ordinary Portland cement (OPC) to evaluate its feasibility as a sustainable alternative.

### Research significance

The research aims to address the optimised use of industrial by-products GGBS and FA, alongside MCK for the development of a one-part alkali-activated binder system. The findings highlight the proportioning for the one-part high-performance with optimised use of industrial by-product for achieving a low-global warming potential binder system to promoting a circular economy model. Inclusion of MCK, characterised by a high surface area, as a precursor not only boosts the reactivity of the cementitious binder but also enhances the pore-filling effect. Use of BBD-based response surface methodology enables robust statistical optimisation. BBD enhances confidence in performance prediction and offers practical guidance for scaling one-part alkali-activated binders for engineering applications. The proposed binder exhibits a lower environmental footprint, reducing reliance on cement and associated carbon emissions. The outcomes of this research contribute to the development of sustainable construction materials by using industrial waste effectively.

## Materials and methodology

### Materials

**Fig. 1:**
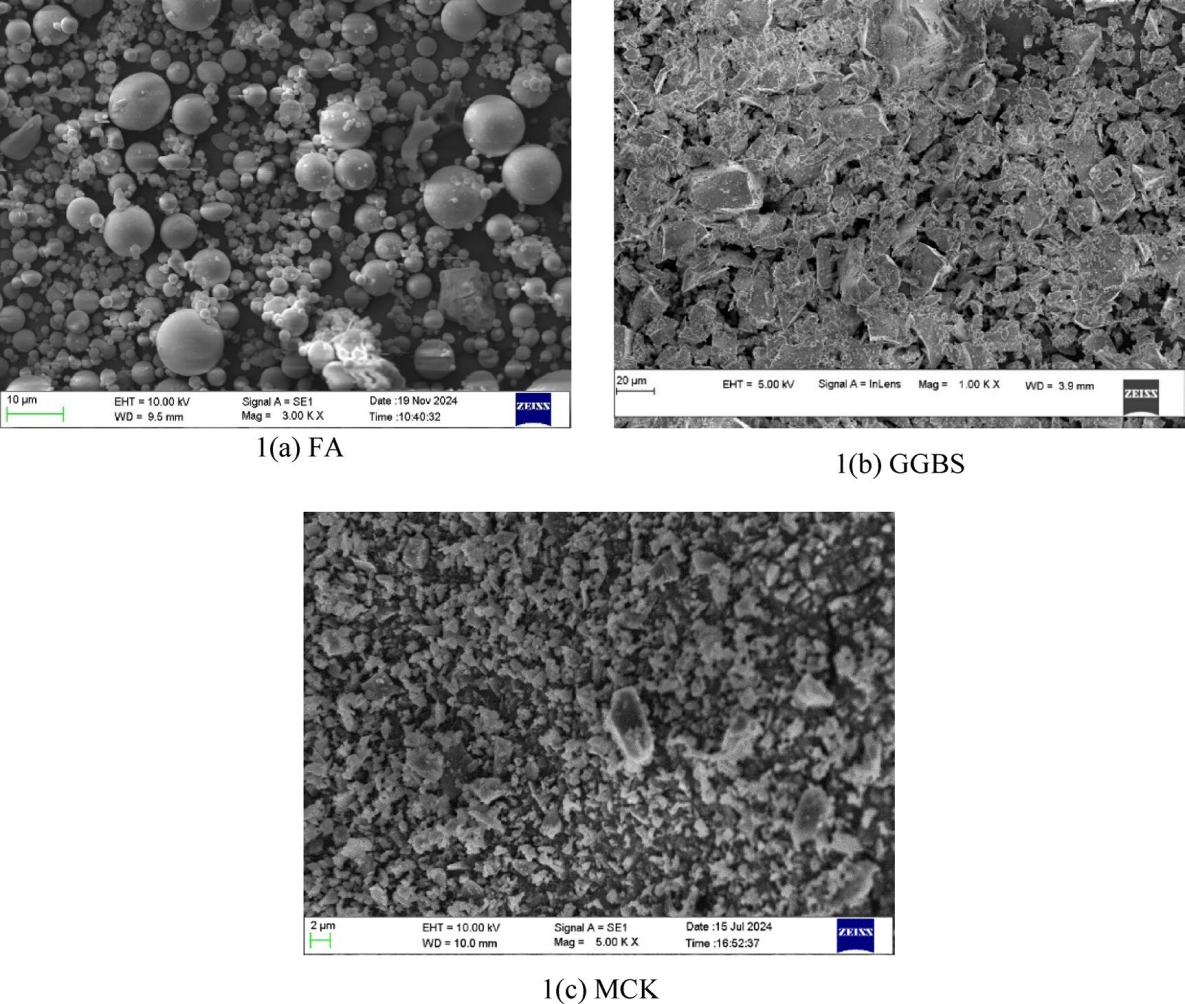
SEM micrographs of FA, GGBS and MCK.

**Fig. 2:**
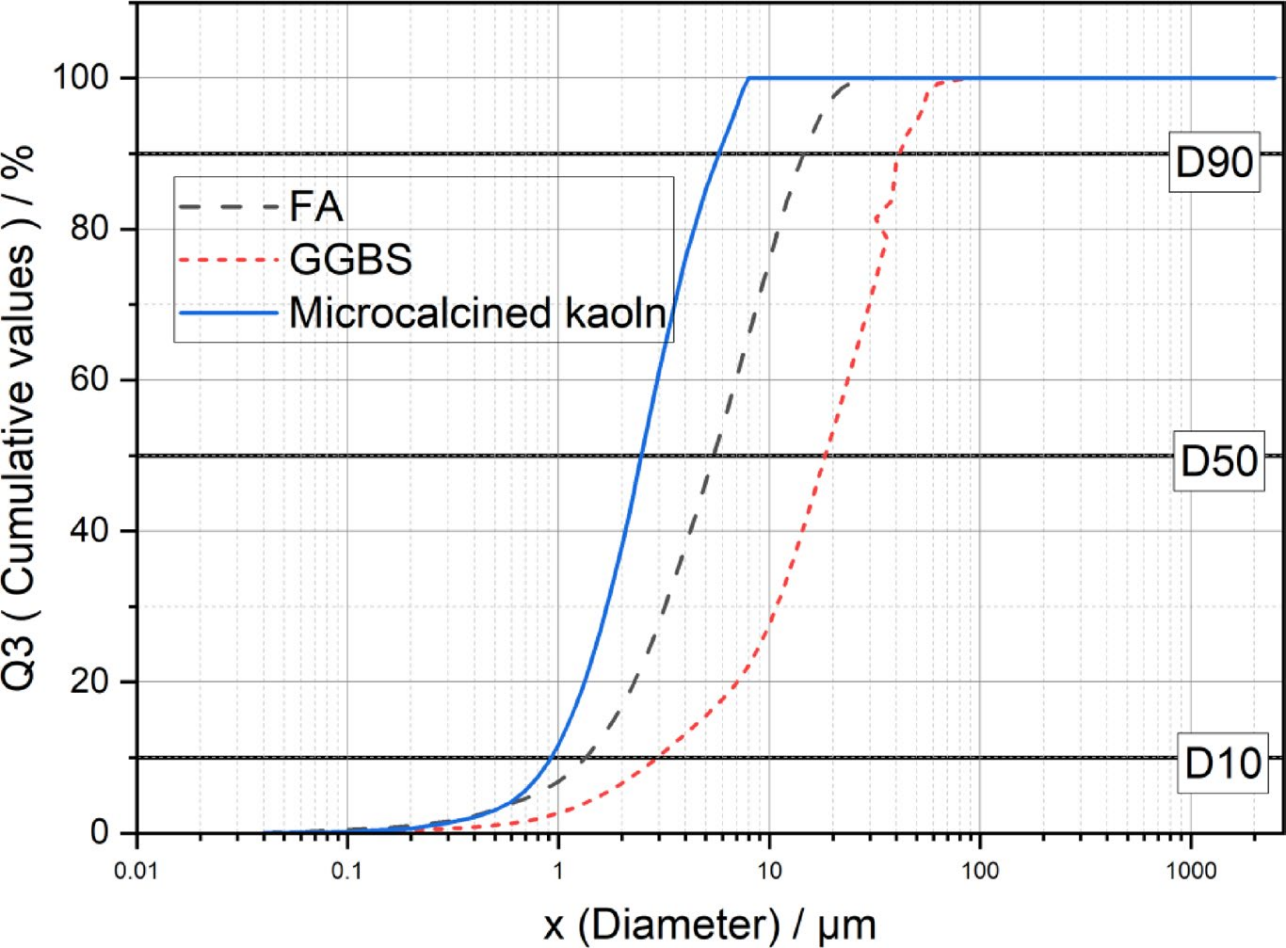
Particle Size distribution of the precursors.

**Fig. 3:**
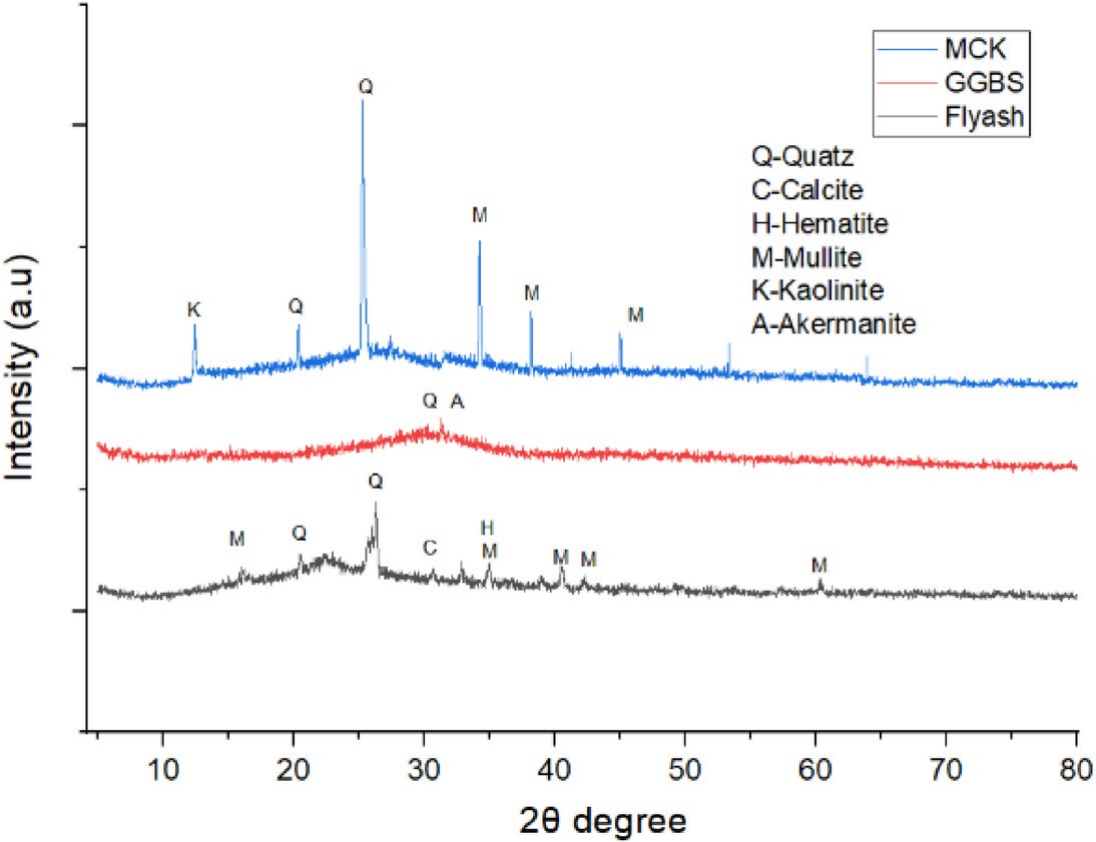
XRD results of precursors.

The present study uses Class F flyash, ground granulated blast furnace slag, and micro-calcined kaolin as aluminosilicate precursors. The surface morphological characteristics are determined using SEM, as illustrated in Figs. 1 (a) - (c). FA is sourced from the Bellary Thermal Power Station, Bellary, India. It exhibits predominantly spherical particles ranging from 0.04 μm to 32 μm Fig. 1(a), aligning with the particle size distribution (PSD) depicted in Fig. 2. Specific gravity of FA is 2.3. The XRD analysis of FA, as presented in Fig. 3, confirmed a largely amorphous structure with crystalline inclusions such as quartz, mullite, calcite, and hematite^4^. GGBS, sourced from JSW Steel in Bellary, India, are found to possess irregular angular particles as seen from Fig. 1 (b). The particle size is distributed between 0.04 μm and 112 μm and the specific gravity of 2.28. Its XRD profile, illustrated in Fig. 3, revealed a predominantly amorphous phase with minor crystalline peaks of calcite and akermanite^39^. MCK, obtained from Astra Chemicals, Chennai, consisted of fine, angular particles with sharp edges, as depicted in Fig. 1 (c), with particle sizes ranging from 0.04 μm to 9 μm. The material had a specific gravity of 2.58 and showed quartz, mullite, and kaolinite phases^19^ in its XRD spectrum presented in Fig. 3. The oxide composition of all three precursors is presented in Table 1. Sodium metasilicate (Na₂SiO₃), also supplied by Astra Chemicals, served as the solid alkali activator. This white, water-soluble inorganic salt had a specific gravity of 2.43 and maintained a Na₂O: SiO₂ molar ratio close to 1:1 (± 0.05). It was used in powdered form to facilitate dry mixing in the one-part binder system.

**Table 1:** Chemical composition of precursors.

Oxides (%)	FA	GGBS	MCK
SiO_2_	53.25	37.30	53.28
Al_2_O_3_	25.62	16.60	37.69
Fe_2_O_3_	6.4	0.37	1.98
CaO	4.7	34.70	0.7
TiO_2_	1.92	0.82	-
MgO	1.04	6.87	0.6
MnO	0.07	0.96	-
Na_2_O	2.22	0.31	0.28
K_2_O	2.05	0.63	0.41
SO_3_	1.29	1.35	0.11
Loss of ignition	1.4	-	4.75
Specific Gravity	2.3	2.28	2.58
Average particle size (µm)	6.87	22.31	2.87

### Optimisation strategy: box-behnken design

**Fig. 4:**
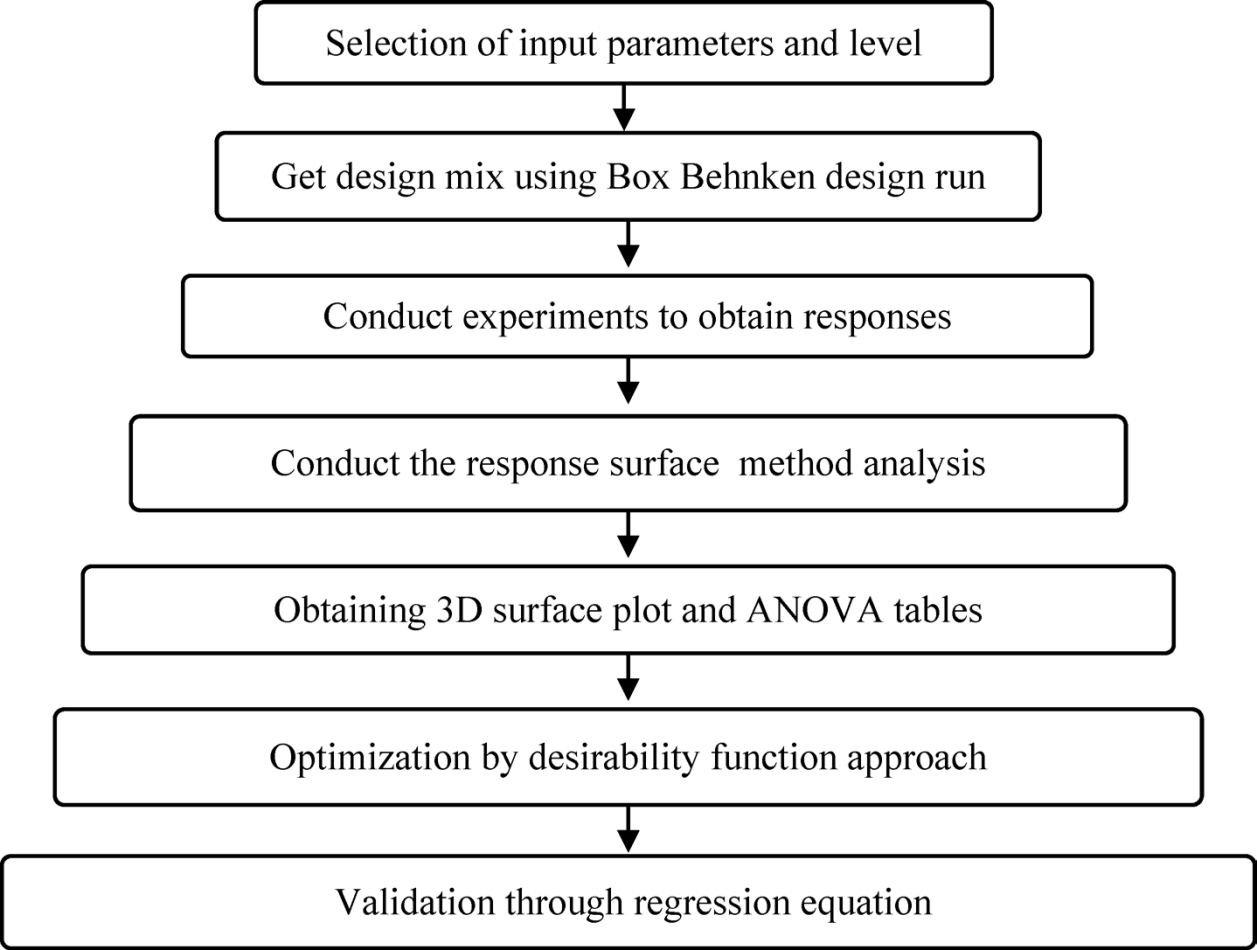
Flow chart illustrating the box behnken design methodology.

For the optimization of the ternary binder system, a Box-Behnken Design (BBD) was carried out using Minitab software 16 version^40^. The experimental design is explained in Fig. 4. Three independent variables, namely FA content (0–25%), MCK content (0–25%), and water-to-binder ratio (0.24–0.30), are chosen based on preliminary experiments aiming for ambient-cured mixtures with compressive strength above 50 MPa and acceptable fresh characteristics. The BBD design required 15 experimental runs, of which three were centre points to evaluate the design’s reproducibility and account for the experimental error. The factor levels are described in Table 2, and the design parameters are listed in Table 3. The mix designs based on the BBD are described in Table 4. For example, mix design F0C0W27 represents a mixture containing 0% FA, 0% MCK, and a W/B ratio of 0.27, which means that the precursor material is 100% GGBS. The GGBS content was kept at a minimum of 50% in all mixes, and the activator content was fixed at 12% in all mixes.

**Table 2:** Level and Factors used in BBD.

Level and Factors used in BBD			
Parameters	Level 1	Level 2	Level 3
Flyash (A)	0%	12.50%	25%
Micro calcined kaolin (B)	0%	12.50%	25%
W/B (C)	0.24%	0.27%	0.3%

**Table 3:** Box Behnken Design runs.

Box-behnken design			
Factors	3	Replicates	1
Base runs	15	Total runs	15
Base blocks	1	Total blocks	1
Center Points	3		

**Table 4:** Mix proportions using BBD-DOE approach.

Mix No.	Mix ID	FlyAsh (%)	MCK (%)	W/B(%)	GGBS (%)
C1	F0C0W27	0	0	0.27	100
C2	F25C0W27	25	0	0.27	75
C3	F0C25W27	0	25	0.27	75
C4	F25C25W27	25	25	0.27	50
C5	F0C12.5W24	0	12.5	0.24	87.5
C6	F25C12.5W24	25	12.5	0.24	62.5
C7	F0C12.5W30	0	12.5	0.30	87.5
C8	F25C12.5W30	25	12.5	0.30	62.5
C9	F12.5C0W24	12.5	0	0.24	87.5
C10	F12.5C25W24	12.5	25	0.24	62.5
C11	F12.5C0W30	12.5	0	0.30	87.5
C12	F12.5C25W30	12.5	25	0.30	62.5
C13	F12.5C12.5W27	12.5	12.5	0.27	75
C14	F12.5C12.5W27	12.5	12.5	0.27	75
C15	F12.5C12.5W27	12.5	12.5	0.27	75

### Specimens preparation and testing methods

The dry components - FA, GGBS, MCK, and sodium metasilicate - are homogenised in a Hobart mixer for one minute. The activator content in the binder is maintained at 12%, as increasing it dosage beyond this limit led to a reduction in compressive strength development^41,42^. Water is then added, followed by low-speed mixing for one minute and high-speed mixing for two minutes at 65 rpm. The fresh paste is cast into PVC moulds (50 mm × 50 mm × 50 mm) and compacted using vibration. Specimens were demoulded after 24 h and cured under ambient conditions until testing. Compressive strength is evaluated at 7 and 28 days using three replicate specimens per mix, following IS 516 (1959)^43^. Flowability is assessed using a flow table in accordance with IS 5512 (1983)^44^. Initial and final setting times are determined using a Vicat apparatus, following IS 4031 (Part 5): 2005^45^.

### Microstructure analysis

To investigate reaction mechanisms and phases, microstructural analyses are carried out on the 28-day cured OP-TAB samples. SEM analysis is performed using a ZEISS EVO MA18 scanning electron microscope equipped with an Oxford EDS system for elemental analysis. Before the analysis, the samples are gold-coated using the sputtering method for better conductivity. The SEM analysis helped in understanding the morphology of the matrix, pore structure, and unreacted particles, while the EDS analysis helped in understanding the elemental composition.

XRD analysis is carried out using a Rigaku Miniflex 600 diffractometer with a voltage of 40 kV and a current of 15 mA. The powdered samples are scanned, and the phases are identified using the X’Pert High Score software version 5.1b^46^. The results from the microanalysis validated the development of reaction products and helped in understanding the mechanical properties of the binder system.

## Results and discussion

### Response surface analysis using BBD DOE

The BBD experimental results of the OP-TAB mixes are presented in Table 5. Fifteen different mix combinations are performed in the study, each with a different FA, MCK level, and water-to-binder ratio (W/B), while keeping the relative proportions of GGBS and the activator constant.

The results showed that flowability is mainly influenced by the W/B ratio, and proportions of precursors have the least significant effect on it. The mix F25C12.5W30 showed the highest flowability (150%), while the mixes with lower W/B ratios, like F0C12.5W24, showed lower flow values (74%). The initial setting time (IST) and final setting time (FST) are highly dependent on GGBS content and W/B ratio. The mixes with higher GGBS content and lower water content, like F0C0W27 and F0C12.5W24, showed the shortest setting times. This is because of the higher calcium content in GGBS, which helps in the quick formation of calcium-alumino-silicate-hydrate (C-A-S-H) gels, thus causing faster hardening.

Regarding mechanical properties, the compressive strength at 7 and 28 days differed among the mixes. The highest 28-day strength (62 MPa) is achieved by mix F25C12.5W24, which balances FA and MCK content with a moderate W/B ratio. This mix demonstrated effective geopolymerisation and matrix densification, validating the synergistic effect of ternary precursor blending.

**Table 5:** BBD-RSM test results of OP-TAB mix.

Mix No.	Mix ID	Flowability (%)	IST (min)	FST (min)	Comp. Strength 7 Days (MPa)	Comp. Strength 28 Days (MPa)
C1	F0C0W27	110	69	90	37	42
C2	F25C0W27	135	120	147	24	52
C3	F0C25W27	124	78	150	32	50
C4	F25C25W27	136	128	225	27	55
C5	F0C12.5W24	74	62	93	41	50
C6	F25C12.5W24	107	99	180	31	62
C7	F0C12.5W30	148	130	210	24	28
C8	F25C12.5W30	150	148	235	22	32
C9	F12.5C0W24	82	68	98	30	48
C10	F12.5C25W24	95	88	122	28	52
C11	F12.5C0W30	140	135	210	28	29
C12	F12.5C25W30	144	155	215	22	32
C13	F12.5C12.5W27	130	110	141	25	46
C14	F12.5C12.5W27	130	112	138	23	45
C15	F12.5C12.5W27	130	113	144	26	42

### Response Surface Plots

**Fig. 5:**
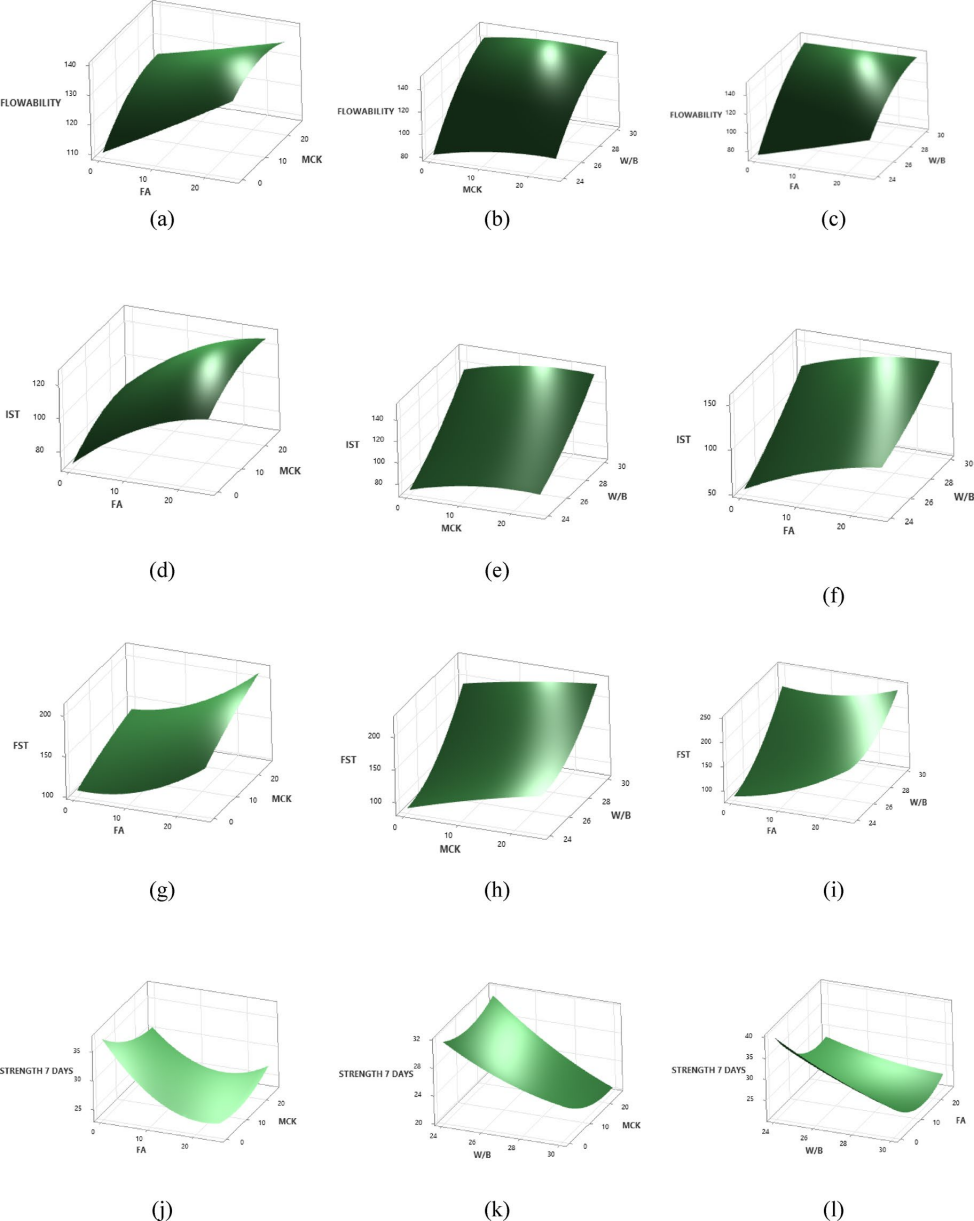

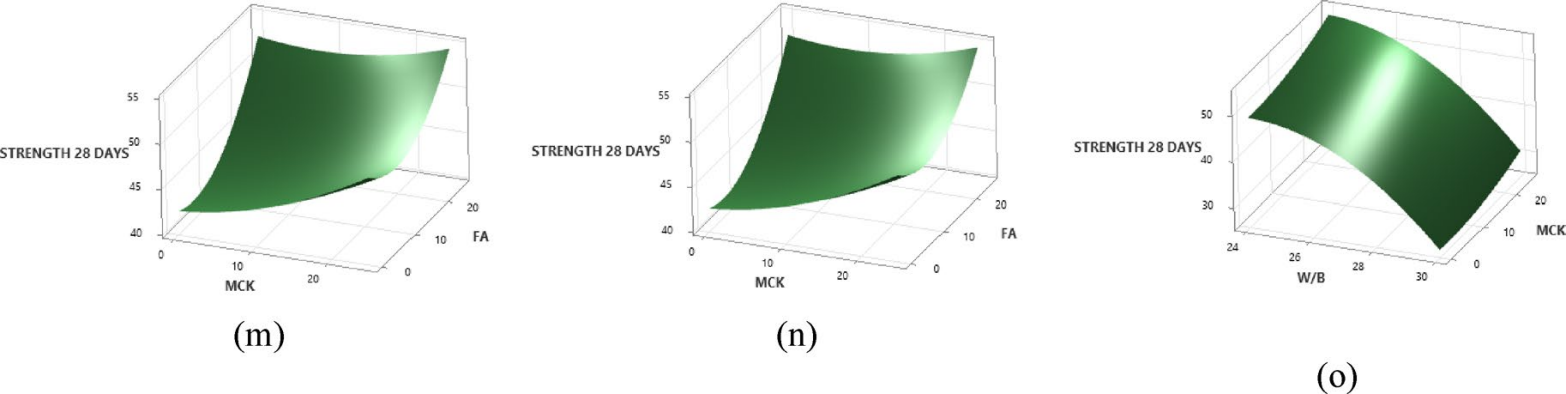
3D response surface plots for flowability, IST and FST and compressive strength of OP-TAB mixes.

Using Minitab software, the 3D response surface plots are generated to visualise the interaction effects among variables. These plots provided insights into how combinations of FA, MCK, and W/B ratio influenced each response parameter. A curved surface indicates significant interaction effects, while a flat surface suggests minimal interaction^33^. All 3D surface plots are generated to evaluate the interaction effects of two variables by keeping one variable constant at level 2. Figure 5 (a – o) illustrates the influence of three key parameters on the response behaviour of the ternary blend slag-based system.

#### **Flowability**

Figure 5(a) shows the effect of FA and MCK on the flow characteristics of ternary OP-TAB. Flow increases by approximately 20–25% as both FA and MCK rise from 0% to 25%. There is a significant increase in flowability when both FA and MCK are increased in the binder. The increase appears to be more pronounced at higher levels of FA, suggesting flyash has a strong influence^27^. The surface morphology and particle shape of the flyash facilitates ball-bearing effect within the binder system, which may contribute to better flowability in the binder system^12,38^. Figure 5(b) illustrates the lowest flow value for maximum MCK and the least w/b ratio, and the maximum flow is found for the least value of MCK and the maximum w/b ratio. This indicates that the primary factor affecting the binder’s flow values is the w/b ratio, compared to MCK dosages. Figure 5(c) indicates that at lower w/b ratios, an increase in FA content results in enhanced flow values. However, at a higher w/b ratio of 0.30, the impact of FA becomes minimal, as indicated by the flat contour. This suggests that flowability is primarily influenced by the w/b ratio, while the contribution of FA becomes less significant at higher w/b levels. Flowability is fundamentally controlled by paste rheology and the availability of free water, which reduces the interparticle friction, resulting into a more significant factor irrespective of the type and proportions of precursors^12^. In contrast, the influence of MCK and FA is indirect and primarily associated with particle morphology, surface area, and reactivity.

#### Initial setting time

Figure 5(d) reveals that the influence of IST increases by approximately 50% when both FA and MCK are at the levels of 25%. However, from the plot, it is clearly evident that the influence of FA delays the setting compared to MCK. FA and MCK are major composed of aluminosilcates with low reactive calcium. In the alkali-activated system, early setting is associated with early dissolution of calcium-rich gels. The presence of reactive aluminates and silicates in MCK dissolves faster for polycondensation compared FA. As shown in Figure 5(e), when MCK content is held constant, variations in the w/b ratio have a substantial impact on IST. In the absence of MCK, increasing the w/b ratio leads to a notable rise in IST. MCK slightly delays setting, likely due to its fine particle size and high-water demand, but its effect is less pronounced than w/b. Figure 5(f) shows that as the FA content increases to 25% gradually, the flow gradually increases with increasing in w/b content. This trend confirms the synergistic effect between FA incorporation and water availability in enhancing workability. FA being less reactive initially delayed reaction mechanism, lowers the initial yield stress and allows the paste to remain workable for a longer duration^30^.

#### Final setting time

Figure 5(g) shows the influence of FST increases by 120% when FA and MCK are at 25%. The minimum value of FST is observed between 0% and 10% of FA, and the maximum value is observed at 25%,. Although both precursors contribute to delayed setting, the response surface indicates that the influence of MCK is slightly higher than that of FA within the studied range. Figure 5(h) reveals that the surface is steeply sloped along w/b, showing this strong effect. Increasing MCK further delays setting, but less significant than W/B. This behaviour can be explained by dilution effects: higher water content reduces ionic concentration in the pore solution, slows dissolution–precipitation reactions, and delays the percolation of C-(A)-S-H and/or N-A-S-H gel networks^27^. Figure 5(i) illustrates that the increasing value of the w/b ratio increases FST values. FA has lower early reactivity than MCK, which also delays setting time, though its effect is less pronounced than w/b^47^. Overall, the results exhibit that both FA and MCK contribute to increased FST through reactivity and surface-area effects, the w/b ratio governs reaction environment, and therefore it is the most dominant influence on final setting behaviour in the ternary OP-TAB system.

####  7 days Compressive strength

The response surface plot of FA and MCK ratio of the OP-TAB mixes on 7 days compressive strength is shown in Fig. 5 (j). The maximum compressive strength value is observed at the lowest value of FA and MCK. Strength drops by about 34% at intermediate FA and MCK levels. The early age strength at lower levels of FA and MCK exhibits dominance of calcium-rich GGBS in polymerisation. In AAB, early strength is achieved by formation of C-A-S-H gel, which gives early rigidity. Figure 5(k) reveals that compressive strength is maximum for the least value of w/b and MCK content. As W/B increases, 7-day strength decreases. This trend is well establish principle with cementitious binder system that, as more water leads to a more porous matrix, reducing strength^3^. The influence of MCK at lower levels appears beneficial for maintaining strength; however, increasing MCK content at higher w/b ratios may further reduce early-age strength due to increased water demand. Figure 5(l) surface plot illustrates strength decreases as FA increases from 0 to 25%. Maximum compressive strength occurs at low FA and low w/b ratio. However, FA improves long-term strength, but it delays early strength gain because its pozzolanic reaction is slower than cement hydration. This is due to excessive replacement of reactive material with slower-reacting pozzolanic materials, which at early ages (7 days) do not contribute much to strength^48^.

#### 28 Days compressive strength

The influence of FA and MCK ratio on the OP-AAB mix 28 days compressive strength is shown in Fig. 5(m). Compressive strength increases by 37.5% as MCK and FA increase from 0 to 25%. Both MCK and FA contribute to the long-term strength development of the binder. The early-age strength is predominantly with calcium-driven reactions. However, the long-term strength development is significantly influenced by the reactivity of FA and the high pozzolanic/geopolymeric potential of MCK. Figure 5(n) demonstrates that maximum compressive strength is at high MCK and low w/b ratio. This is due to MCK enhancing geopolymerization and low water content ensures a denser matrix. Figure 5(o) shows higher strength is achieved with 24% w/b and 25% FA. w/b has a consistent negative effect as expected in a cementitious system^49^. The increased availability of reactive silica and alumina promotes the formation of a more extensive and interconnected N-A-S-H gel network. In the presence of calcium from GGBS, hybrid C-(A)-S-H/N-A-S-H gel structures may form, leading to improved matrix densification and mechanical performance. Simultaneously, a lower w/b ratio reduces capillary porosity and increases solid packing density, resulting in a compact and stronger microstructure^38^.

### ANOVA results

The experimental results are statistically analysed using the software *Minitab* to identify the adequacy, reliability, and significance of the developed response surface model. ANOVA is employed to assess the correlations and influences of variables, including actual factor levels, responses, and their two-way interactions.

**Fig. 6:**
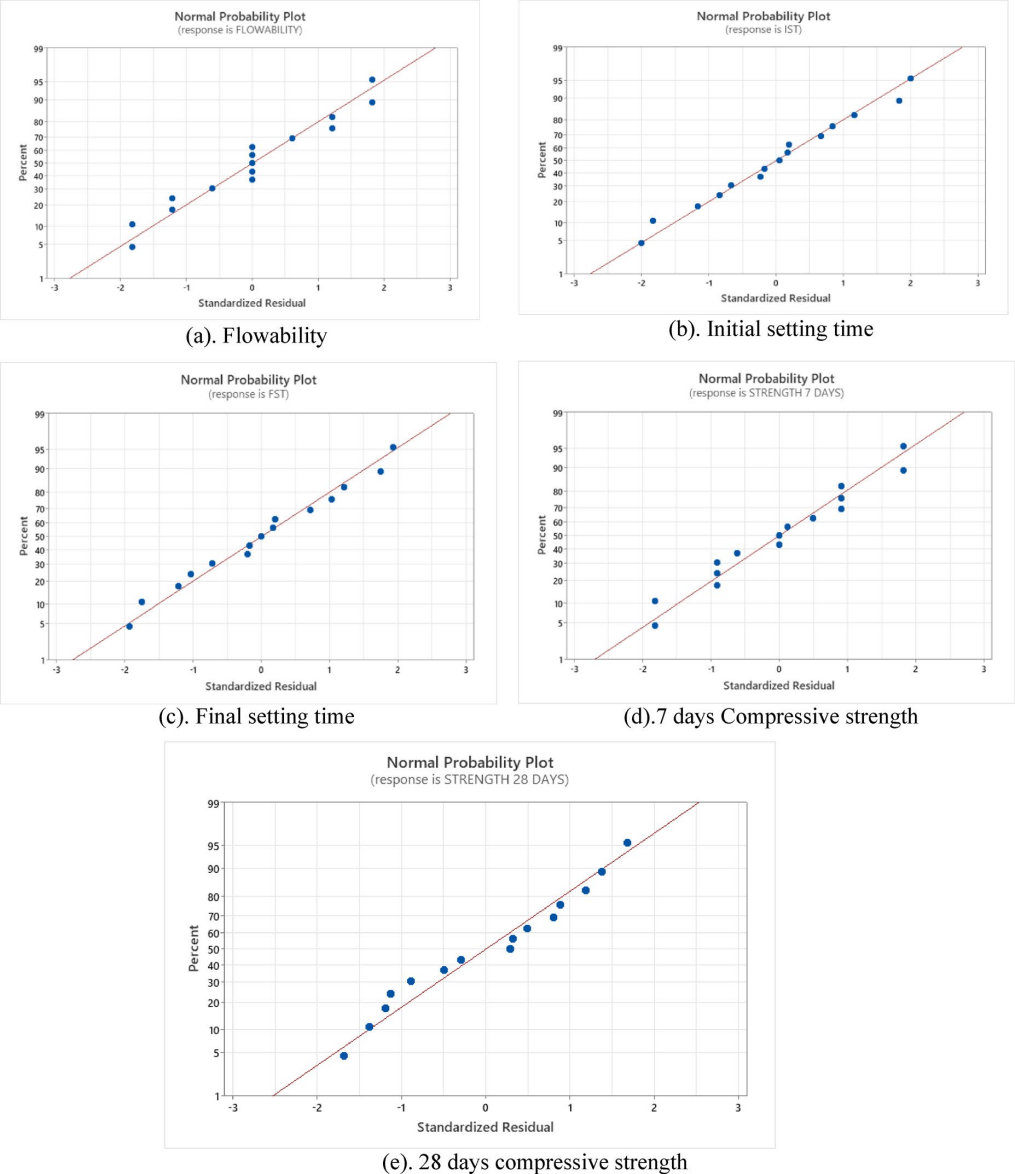
Normal plot of standardised residual response models of ternary blend slag-based OP-TAB mix.

The p-values of all models are not consistently below 0.05, indicating that not all factors are statistically significant at the 95% confidence level, as shown in Table 6. The 7-day compressive strength model shows marginal significance (*P* = 0.079), suggesting a comparatively weaker influence of the selected factors at early ages. Early-age compressive strength play vital role in formwork handling and construction scheduling. In alkali-activated binder systems, early-age strength development is associated with higher variability due to evolving reaction kinetics and curing sensitivity, which can reduce statistical power without negating engineering relevance. The R² values, which reflect the correlation between predicted and actual responses, are presented in the same Table 6. Higher R² values for flowability, and compressive strength at 28 days 99.83%, and 97.79%, respectively, indicate strong agreement between predicted and observed values, suggesting a good model fit. For initial, final setting times, and 7-day compressive strength the R² values of 96.96%, 95.08% and 87.16% shows moderate level of agreement between predictions and actual results. The data distribution and adequacy are verified using normal probability plots, as shown in Fig. 6. The residuals of all dependent variables lie approximately along a straight line, confirming the normal distribution of data. Table 7 further highlights the p-value obtained through ANOVA for main factors, their interaction and quadratic terms with respect to how flowability, compressive strength, and setting time.

**Table 6:** ANOVA response models results analysed through BBD-RSM.

Model	Responses				
	Flowability	IST	FST	Comp str 7d	Comp. str 28d
Standard deviation	1.64317	8.61878	18.3807	3.29140	2.52653
Mean	130.0	111.67	141.0	24.67	44.33
R^2^	99.83%	96.96%	95.08%	87.16%	97.79%
F value	61.9	17.73	10.73	3.77	24.53
P value	< 0.001	0.003	0.009	0.079	0.001
lack of fit	0.016	0.019	0.016	0.126	0.378

Table 7 depicts p-values at 95% confidence level (*p* < 0.05), the statistical significance of each parameter can be interpreted as follows. For flowability of the OP-TAB, the factor influence follows the order: C > A > B > AC AB > BC. Flowability is primarily governed by w/b ratio and FA content, supported by strong statistical evidence. Among interaction terms, AC (FA × w/b) is highly significant, confirming the synergistic effect between FA morphology and water availability in improving flow. For initial setting time, the order is C > A > B > AC > AB > BC, whereas for final setting time it is C > A > B > AC > BC > AB. The dilution effect due to w/b and the slower reactivity of FA dominate the initial setting behaviour. A similar pattern is observed in FST behaviour with the influence of both precursor chemistry and dilution effects. For compressive strength at 7 days, the order of influence is C > A > B > AB > AC > BC. W/B and FA are statistically significant factors for the early age strength. However, none of the interaction or quadratic terms are significant which exhibits its mainly depends on calcium-rich precursor sources. Similarly, for compressive strength at 28 days, the influence order is C > A > B > AC > AB > BC. Interaction terms remain statistically insignificant for long-term strength. It is primarily governed by individual contributions of FA and w/b rather than their combined interactions.

**Table 7:** **p**-**value obtained through ANOVA for the response factors and their interactions**.

Model	Responses				
	Flowability	IST	FST	Compressive strength (7 days)	Compressive strength (28 days)
A= flyash	< 0.0001	0.001	0.005	0.023	0.007
B = MCK	0.001	0.067	0.024	0.332	0.053
C = w/b%	< 0.0001	< 0.0001	0.001	0.015	0.000
AB	0.011	0.956	0.645	0.279	0.368
AC	< 0.0001	0.321	0.152	0.279	0.174
BC	0.041	1.000	0.627	0.570	0.851
A^2^	0.679	0.163	0.175	0.071	0.027
B^2^	0.005	0.268	0.757	0.446	0.357
C^2^	< 0.0001	0.281	0.058	0.615	0.009

In multi-objective optimisation, multiple responses must be optimised simultaneously, which often have conflicting requirements. Improving one response may adversely affect the performance of another. To address this challenge, the desirability function approach is used within Response Surface Methodology (RSM). The desirability function converts each response into a dimensionless scale ranging from 0 to 1. The aim of optimisation is to achieve the desirability functions as close to 1 as possible. Each response (e.g., compressive strength, flow, IST, FST) is first transformed into an individual desirability value depending on the optimisation criteria defined in Table 8. All responses are assigned with the maximum goal for optimisation. The higher weightage is given to compressive strength factors, keeping its importance in the durability of binder system.

**Table 8:** Optimisation criteria adopted for desirability function.

Response	Goal	Lower	Target	Upper	Weight	Importance
28 d compressive strength (MPa)	Maximum	32.00	50.00	62.00	1.25	1.25
7 d compressive strength (MPa)	Maximum	22.00	41.00	41.00	1.25	1.25
FST(min)	Maximum	93.00	235.00	235.00	1.00	1.00
IST(min)	Maximum	62.00	155.00	155.00	1.00	1.00
Flowability %	Maximum	74.00	150.0	150.0	1.00	1.00
**Variable Ranges**						
**Variable**	**Goal**			**Values**		
Flyash %	In Range			(0, 25)		
Micro calcined kaolin %	In Range			(0, 25)		
W/B %	In Range			(0, 25)		

**Fig. 7:**
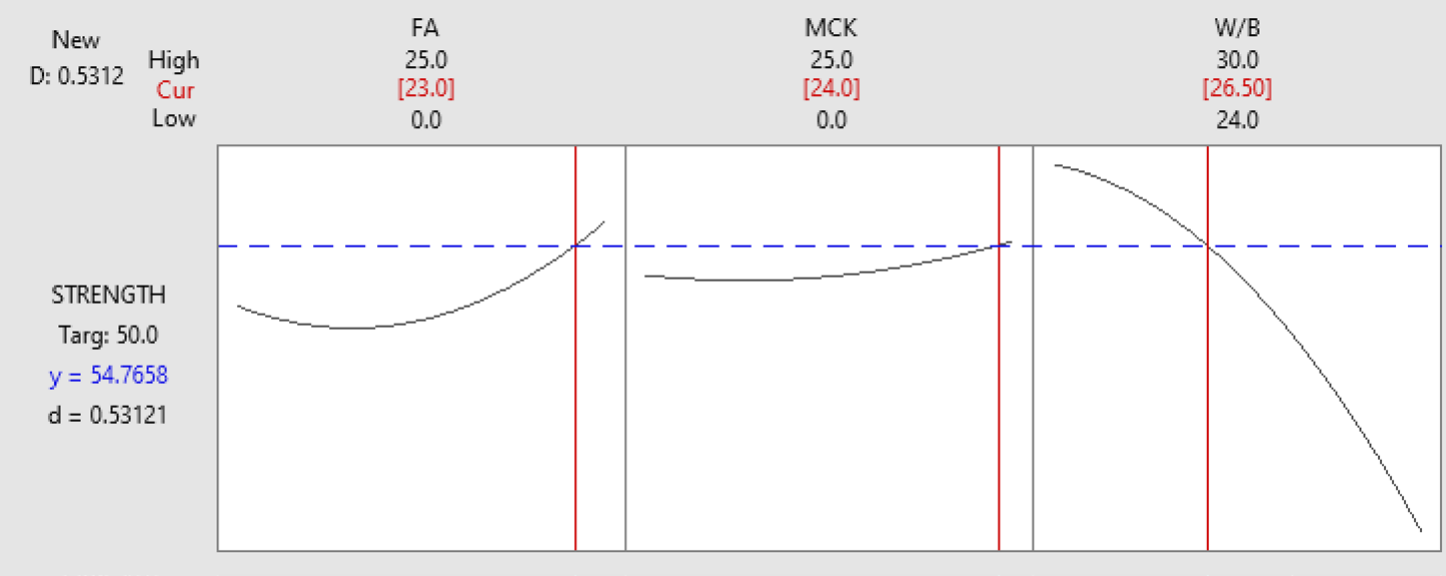
Optimisation ramps.

The selection of parameters is based on a balanced optimisation strategy by prioritising strength, along with sufficient setting time and workability, to maintain practical applicability. The desirability function thus provides a rational compromise solution rather than extreme optimisation of a single parameter. The optimal mix is determined as 23% FA substitution, 26.5% w/b ratio, and 24% MCK. This optimised mix achieved a flowability of 130.12%, initial and final setting times of 114.58 and 186.47 min, respectively, and compressive strengths of 28.12 MPa at 7 days and 55.45 MPa at 28 days, respectively. The optimised combination obtained at a desirability function value of 0.5312, indicating a moderate level of reliability in the predicted results. The results of the optimised mix proportions effectively balance the targeted properties of the binder^3,50^. The relationship between the independent variables and the responses was visualised. These optimisation ramps are presented in Fig. 7 for better interpretation.

### Microstructure and mineralogy

#### SEM and EDS analysis

**Fig. 8:**
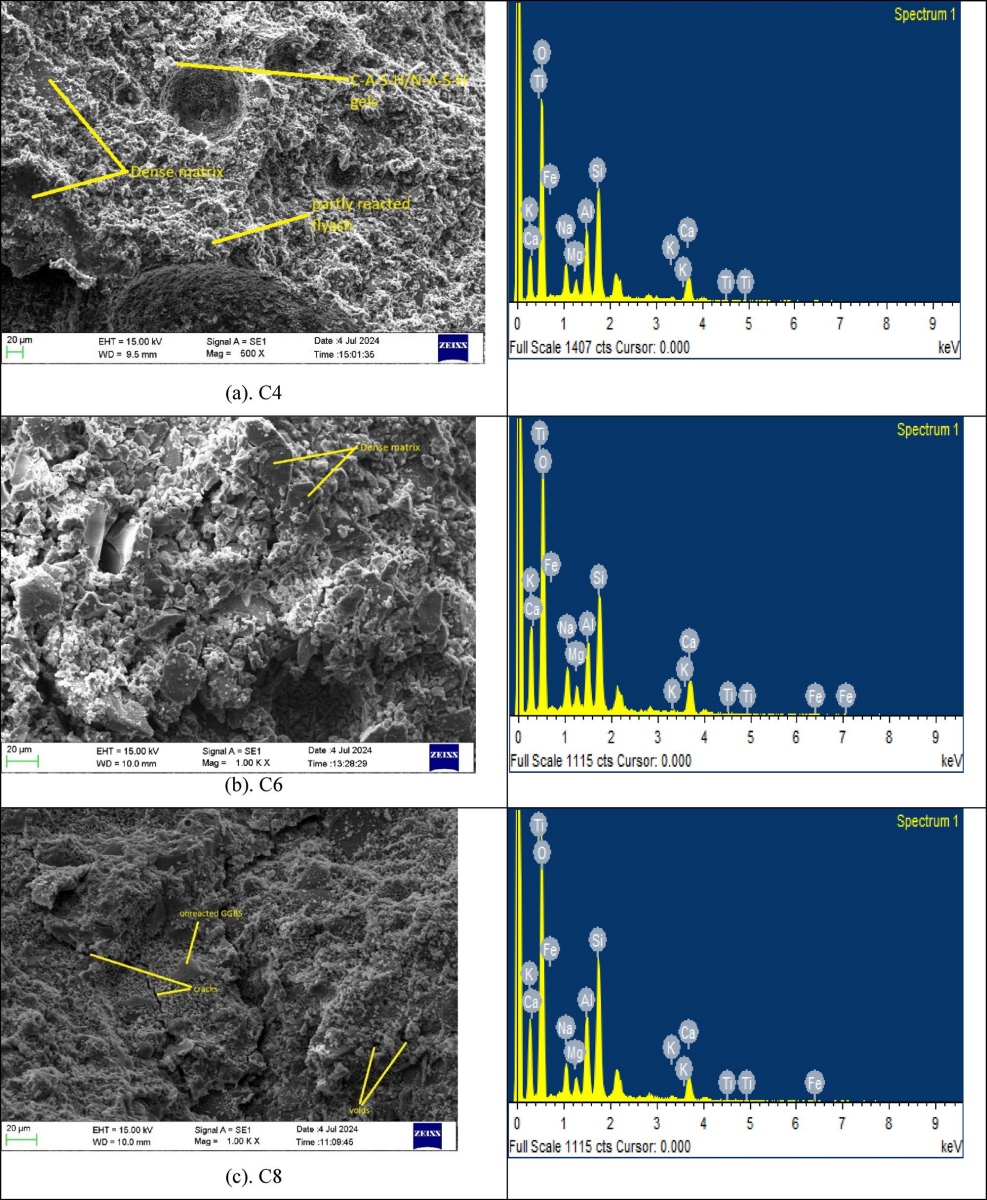

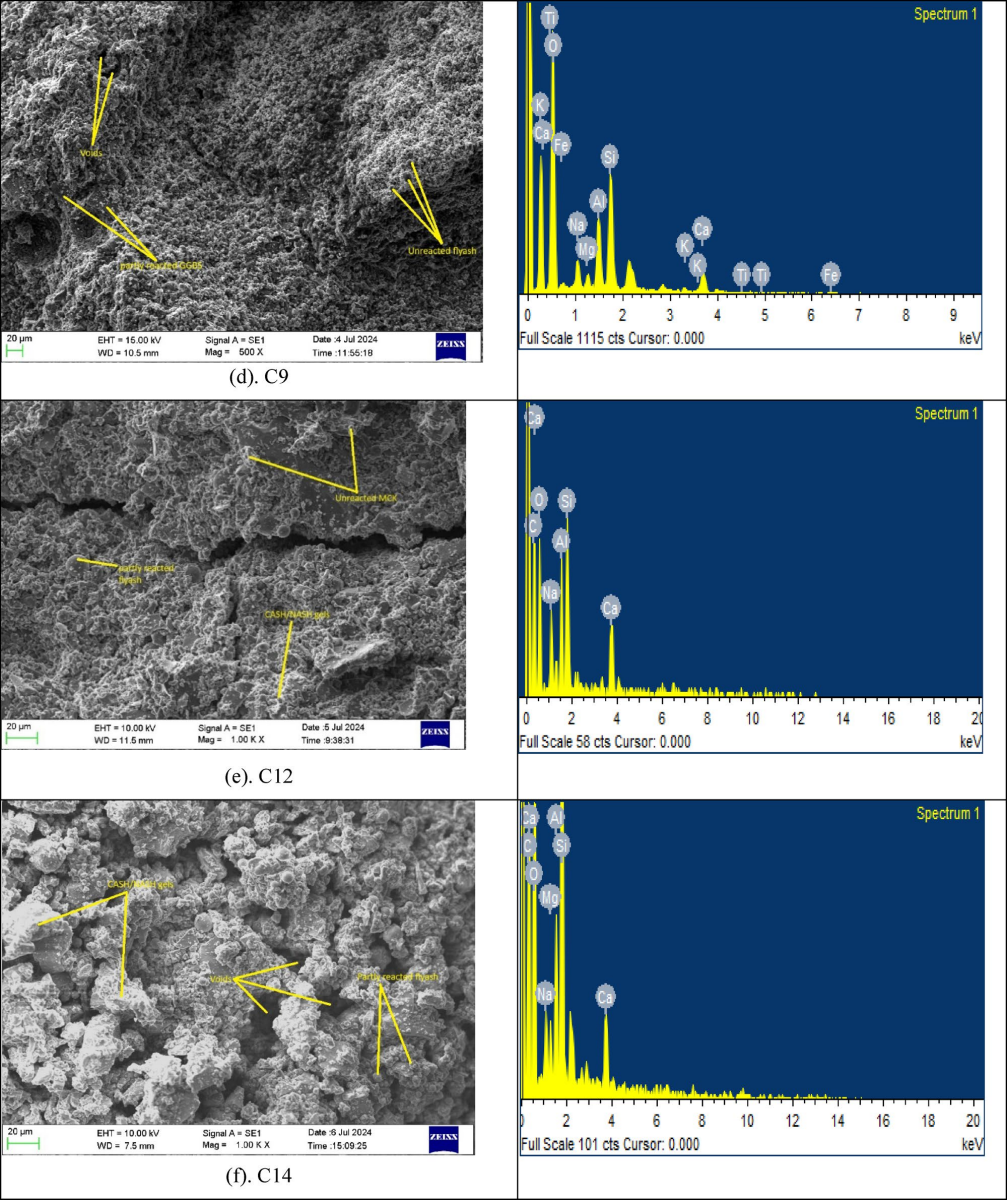
SEM and EDS images (a) C4 (b) C6 (c) C8 (d) C9 (e) C12 and (d) C14.

Figure 8 shows SEM micrographs of six different mixes, selected based on their compressive strength levels at the range high, moderate, and low. Micrographs of all mixes show continuous gelatinous structures throughout their microstructure formation, primarily consisting of C-A-S-H and/or N-A-S-H gels, which are key contributors to strength development in the binder^51,52^. Figure 8(a) and 8(b) shows micrographs of mixes C4 and C6 exhibit significantly denser microstructures compared to the other micrograph. The well-formed, continuous gels suggest a higher degree of polymerisation between the activator and precursor materials, resulting in enhanced compressive strength^29,53^. In contrast, mixes C8 and C12 reveal a higher concentration of voids and micro-cracks, indicating poor gel formation. This is likely due to insufficient activator dosage, which leads to weaker polymer networks and lower strength. Mixes C9 and C14 micrographs show fewer pores and more uniform gel phases than C8 and C12, but less dense than C4 and C6. These types of microstructural formations are consistent with their moderate compressive strength. SEM observations are further correlated with the findings from EDS and XRD analyses.

**Table 10:** EDS results of mixes C4, C6, C8, C9, C12 and C14.

Mix id	Si	Al	Mg	Na	Ca	Ca/(Si + Al)	Na/(Si + Al)	Ca/Si	Ca/Al	Al/Si
C4	16.60	7.94	1.56	4.01	12.17	0.49	0.16	0.73	1.53	0.47
C6	16.37	8.21	1.28	3.15	8.53	0.34	0.12	0.52	1.03	0.50
C8	14.16	6.73	2.11	4.53	13.34	0.63	0.21	0.94	1.98	0.47
C9	16.05	8.76	1.43	3.19	9.24	0.37	0.12	0.57	1.05	0.54
C12	15.78	7.65	1.31	3.21	10.29	0.44	0.13	0.65	1.34	0.48
C14	14.23	6.53	1.24	4.27	8.78	0.42	0.20	0.61	1.34	0.45

Table 10 shows EDS of the elemental composition of mixes C4, C6, C8, C9, C12 and C14. The results are analysed to assess their chemical characteristics and molar ratios. The Si content ranges from 14.16% to 16.95%, Ca content from 8.53 to 13.34 range, and the Al content ranges from 6.53% to 8.76% across the selected mixes. C4 and C6 exhibit moderate Ca/Si ratios of 0.73 and 0.52, respectively, along with balanced Al/Si ratios from 0.47 to 0.50. The Ca/(Si + Al) results exhibit the formation of a stable hybrid N-(C)-A-S-H gel network rather than an excessively calcium-dominated system^54^. This balanced gel chemistry promotes matrix densification and reduces microstructural discontinuities evident from SEM micrographs, thereby enhancing compressive strength. However, C6 exhibits higher compressive strength compared to C4, which shows a balanced calcium-aluminosilicate gel formation^9^. C9 exhibits a relatively higher Al/Si ratio (0.54), denoting increased aluminosilicate cross-linking. However, its Ca/Si (0.57) and Ca/(Si + Al) (0.375) ratios suggest moderate calcium participation. C14 shows slightly higher Na incorporation (Na/(Si + Al) = 0.20), which may accelerate dissolution but can also induce shrinkage-related microcracking if not well balanced^38^. C8 has the highest Ca/Si (0.94) and Ca/(Si + Al) (0.63) ratios, indicating excessive calcium dominance. Although higher calcium can accelerate reaction kinetics, an overabundance may lead to rapid gel precipitation, non-uniform structure formation, and microcrack development. This is supported by the observed surface cracking in SEM images. Similarly, C12 exhibits moderate Ca/Si (0.65) but may have experienced incomplete polymerisation or uneven gel distribution, contributing to increased void content and reduced load-bearing capacity.

#### **X-ray Diffractometer Test**

**Fig. 9:**
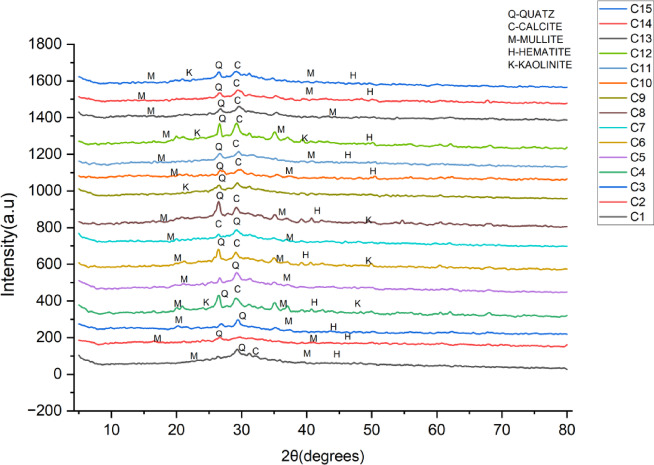
XRD result of OP-TAB mixes.

Figure 9 shows the XRD results of mixes C1 to C15. The results show unique crystalline phases present in the samples. Across all mixes, the main crystalline phases identified are Quartz (Q) prominent peaks at ~ 26–27° 2θ and Kaolinite (K) minor peaks at ~ 12° and ~ 25° at 2θ.

The presence of quartz and calcite is prominent in all mixes, which is an indication that mixes are dominant in the crystal matrix. Mullite is present in all mixes and is constant in the patterns. which is an indication that it is stable in the system. Hematite and kaolinite are present in a few mixes at lower intensities, which is an indication that they are minor phases^55^. The total peak intensities differ slightly between the samples, which could be attributed to differences in composition or amounts of crystalline versus amorphous phases. The stronger mixes (C4, C6) are associated with higher calcite and lower unreacted mullite or kaolinite. Quartz is present in all mixes, but it is not a direct measure of strength, as it is primarily a measure of unreacted silica. Samples such as C9 have broader peaks, which suggest a combination of amorphous and crystalline phases. The lower strength samples (C1, C2, C8) have higher mullite and hematite, which suggest higher amounts of crystalline phases and lower amounts of gel formation^51^.

### Sustainability and cost assessment of the proposed mix

The comparative assessment of 100 g of OPCB and OPTAB, based on Embodied Energy (EE), CO_2_ emission (CO_2_E), Global Warming Potential (GWP), and cost, was performed using a cradle-to-gate framework^56^. This involved estimating the aforementioned parameters from the procurement of raw materials for binder preparation to its production. The values of the multiplicative coefficients for the binder ingredients considered for the ecological and economic assessments are tabulated in Table 11.

**Table 11:** Coefficient used for Sustainability attributes.

Constituent materials	Co-efficient			
	Embodied Energy (kJ/kg) ^57^ ^[,58^ ^[,59^ ^[,60^ ^[,61^	CO_2_ Emission coefficients (Kg-CO_2_/kg) ^62^ ^[,63^ ^[,64^ ^[,65^ ^[,66^	GWP coefficients kgCO_2_eq/kg ^67,]^ ^68^ ^[,69^ ^[,70^	Unit cost Rs/kg ^58^ ^[,65^ ^[,66^ ^[,71^ ^[,72^ ^[,73^ ^[,74^ ^[,75^
Water	0	0	0.001	0
FA	0.04	0.005	0.00526	0.002
GGBS	0.31	0.012	0.0083	0.0046
MCK	3	0.0625	0.42	0.086
OPC	4.5	0.951	0.95	0.009
Sodium Meta Silicate	5.37	0.73	1.11	0.1

**Fig. 10:**
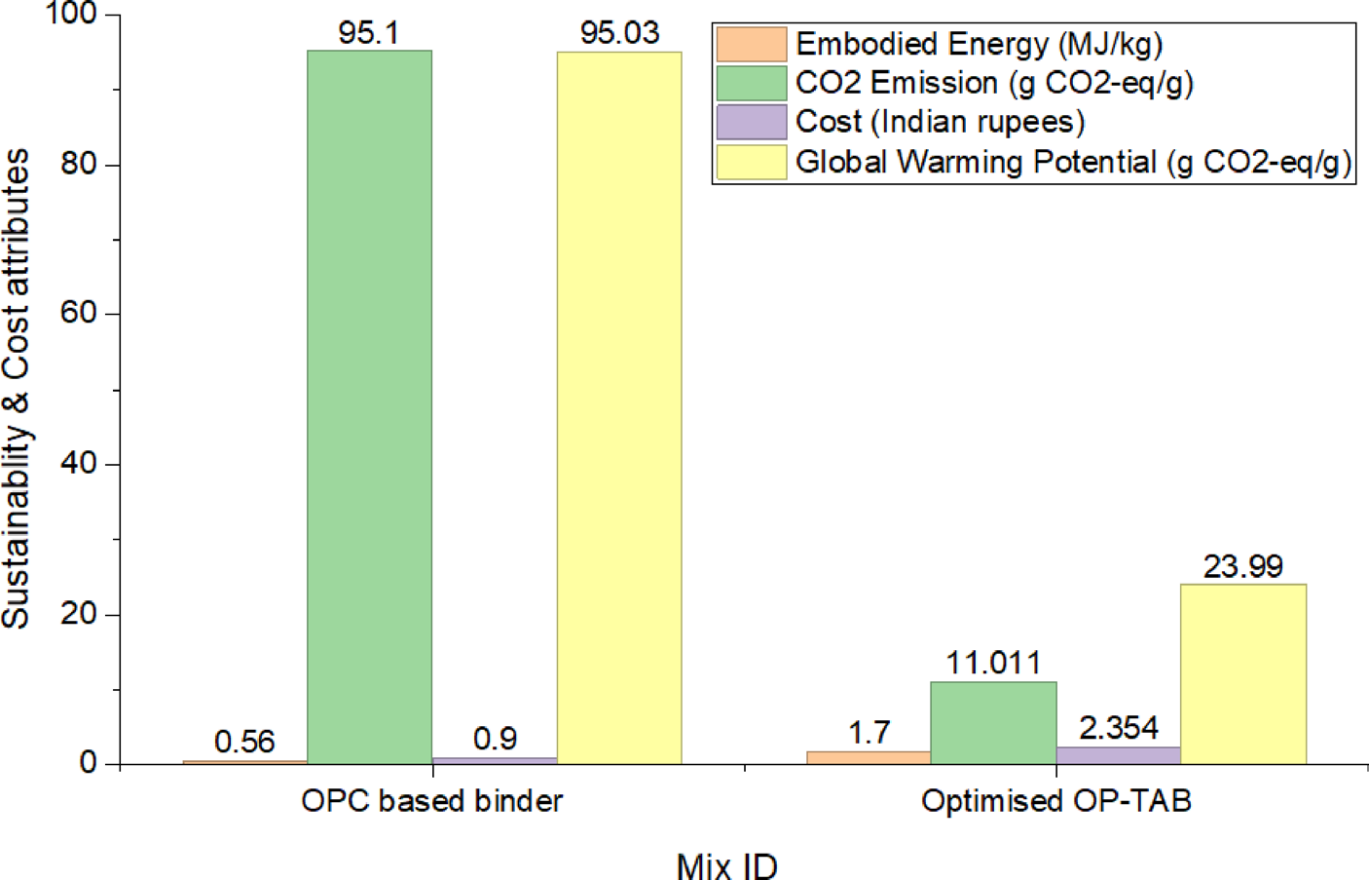
Embodied Energy (EE), CO_2_ emission (CO_2_E), Global Warming Potential (GWP) and cost comparison of OPC and optimised OPTAB mixes.

From Fig. 10, it is evident that the OPCB exhibits a significantly lower EE (0.56 MJ/kg) compared to the optimised OPTAB (1.7 MJ/kg). This indicates that the production and processing of OPTAB require higher energy input, likely due to additional precursor materials such as MCK (3 MJ/kg), as well as the preparation of Sodium Meta Silicate (5.37 MJ/kg). Despite this higher energy demand, EE alone does not fully capture environmental sustainability.

However, CO_2_ emissions of OPCB and optimised OPTAB, when examined found to be contrasting. OPCB shows a high CO_2_ emission of 95.1 g-CO_2_/g, while optimised OPTAB emits only 11.01 g-CO_2_/g, amounting to nearly an 88% decrease. This significant reduction can be attributed to the elimination of clinker production, which is the primary source of CO_2_ emissions in OPCB. The use of industrial by-products and supplementary cementitious materials in OP-TAB plays a crucial role in minimising CO_2_ emissions. This approach makes it an environmentally favourable alternative despite its higher EE.

A similar trend is also noticed in GWP. The GWP of OPCB is 95.03 gCO_2_-eq/g, whereas optimised OP-TAB indicates 23.99 gCO_2_-eq/g. This indicates a reduction of almost 75% in GWP, thus reiterating the better environmental performance of the alkali-activated binder, thereby indicating that the mitigation of greenhouse gases is efficiently done by the OP-TAB system.

However, the Cost analysis indicates that OPTAB is more than twice as expensive as OPCB. OPCB is Rs. 0.9 per unit, whereas optimised OP-TAB is Rs. 2.4. This could be because of the cost of alkali activators. Thus, although OP-TAB performs better in terms of environment, its economic viability could be a challenge for its mass adoption unless cost optimisation techniques are employed.

**Fig. 11:**
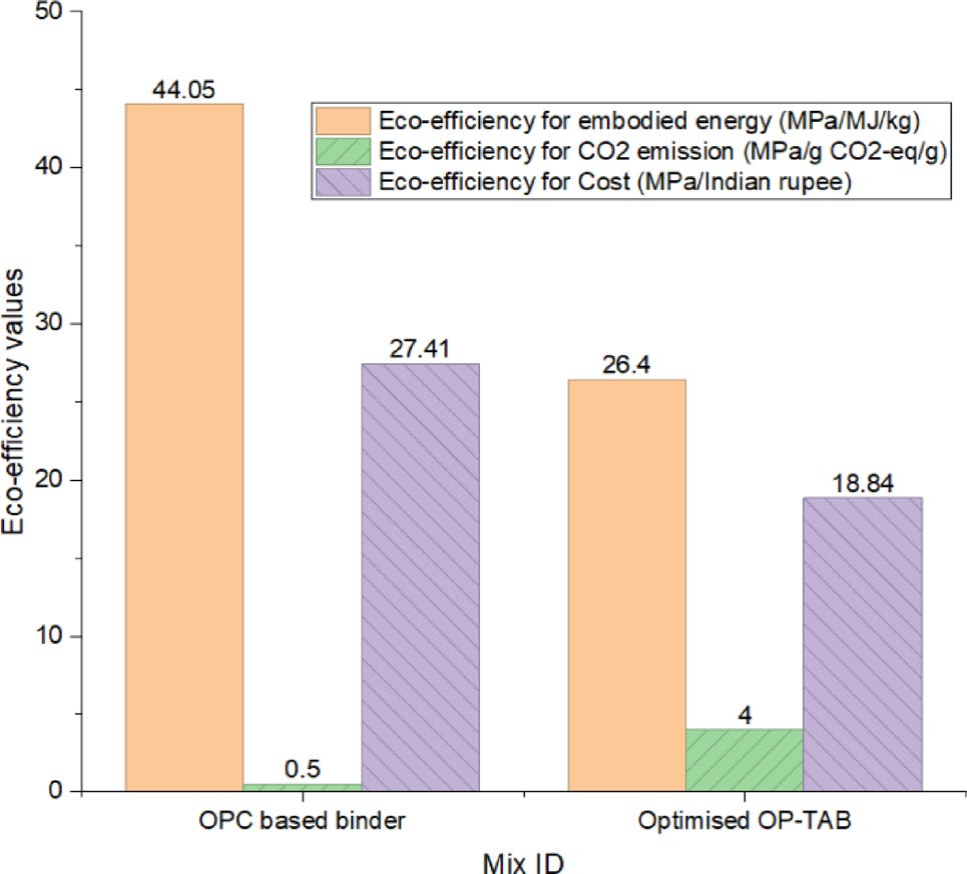
Eco-efficiency indices of OPC and optimised OPTAB mixes.

The eco-efficiency indices, which link the compressive strength with the environmental and ecological effects, is shown in Fig. 11. The eco-efficiency w.r.t. EE shows that the OPCB has a value of 44.05 MPa/MJ/kg, which is much higher than the optimised OP-TAB of 26.4 MPa/MJ/kg. This clearly shows that OPC has better energy efficiency as it provides higher strength for the same amount of energy used.

The eco-efficiency w.r.t CO_2_ emission of OPCB is very low, with a value of 0.5 MPa/gCO_2_-eq/g, while the optimised OP-TAB shows a remarkable improvement of 4 MPa/gCO_2_-eq/g. This eightfold improvement clearly indicates that OP-TAB is significantly more efficient in terms of strength produced per unit of carbon released, thus emphasising its superior environmental benefit in terms of carbon footprint reduction. The eco-efficiency in terms of cost of OPCB is 27.41 MPa/Rs, while that of OP-TAB is 18.84 MPa. The reduced cost efficiency of OP-TAB indicates the increased material and processing cost of alkali activation.

Analysis of both cost and environmental aspects clearly indicates that there is a clear trade-off between the two materials. OPCB is clearly the cheaper material and requires less energy input in its production process, but it has a significantly higher environmental impact, especially in terms of carbon emissions and its effect on global warming. OP-TAB, on the other hand, shows a remarkably better environmental profile, with significantly lower emissions and a reduced effect on the climate.

In contemporary construction practices, materials such as OPTAB may better support long-term environmental goals, even if their upfront expenses are higher. With continued technological goals, the disparity between financial cost and ecological performance could narrow. Streamlining production methods for materials like OPTAB or implementing policy incentives for low carbon alternative could enhance affordability and encourage broader industry acceptance.

## Conclusion

1. The quest for a sustainable industrial-grade binder that can cure and harden at ambient conditions necessitated the use and optimal proportioning of ternary blends of GGBS, FA and MCK. The BBD-RSM has meticulously analysed the influencing parameters such as precursor proportioning and w/b ratio for optimising the key performance indicators that define its applicability, including setting, flowability and strength development.
2. Surface plots and ANOVA suggested that the setting characteristcs is majorly influenced by the w/b ratio and the FA content in the mix. Early age strength depends on proportions of GGBS in the binder, while the long-term strength is influenced primarily by the FA and MCK content, which contributed to the formation of C-NASH, validated through elemental analysis.
3. Based on BBD-RSM optimisation, the optimal mix proportions for the OP-TAB binder are identified as 23% FA, 24% MCK, w/b ratio of 26.5% and 53% GGBS with 12% activator. The results exhibit a flowability of 130.12%, IST of 114.58 min, and FST of 186.47 min, along with compressive strengths are 28.12 MPa and 55.45 MPa at 7 days and 28 days, respectively.
4. The blends of GGBS-FA-MCK mixes with a lower w/b ratio resulted in the formation of a dense and compact matrix. SEM micrographs revealed a compact microstructure with minimal unreacted precursors, which corresponded with the superior mechanical strength achieved.
5. On comparison with the conventional OPC binder, the OPTAB found have higher embodied energy, however significantly lower carbon emission (85% lower than OPCB) and GWP (75% lower than OPC). Though sustainable, the OPTAAB is associated with higher cost, which is mainly due to the activator and transportation of precursors. However, bulk manufacturing units near the source of precursors and waste-derived activators may be a way forward to work on go to market.

### Future recommendation

Future studies should focus on evaluating long-term durability aspects such as shrinkage behaviour, efflorescence resistance, and field-scale performance validation, along with life-cycle cost optimisation through alternative activator development and industrial-scale production strategies.

## Data Availability

The datasets generated during and/or analysed during the current study are available from the corresponding author on reasonable request.
